# Challenges With Developing Secure Mobile Health Applications: Systematic Review

**DOI:** 10.2196/15654

**Published:** 2021-06-21

**Authors:** Bakheet Aljedaani, M Ali Babar

**Affiliations:** 1 Centre for Research on Engineering Software Technologies School of Computer Science The University of Adelaide Adelaide Australia; 2 Computer Science Department Aljumum University College Umm Alqura University Makkah Saudi Arabia; 3 Cyber Security Cooperative Research Centre Adelaide Australia

**Keywords:** systematic literature review, mHealth apps, secure apps, developers, security knowledge

## Abstract

**Background:**

Mobile health (mHealth) apps have gained significant popularity over the last few years due to their tremendous benefits, such as lowering health care costs and increasing patient awareness. However, the sensitivity of health care data makes the security of mHealth apps a serious concern. Poor security practices and lack of security knowledge on the developers’ side can cause several vulnerabilities in mHealth apps.

**Objective:**

In this review paper, we aimed to identify and analyze the reported challenges concerning security that developers of mHealth apps face. Additionally, our study aimed to develop a conceptual framework with the challenges for developing secure apps faced by mHealth app development organizations. The knowledge of such challenges can help to reduce the risk of developing insecure mHealth apps.

**Methods:**

We followed the systematic literature review method for this review. We selected studies that were published between January 2008 and October 2020 since the major app stores launched in 2008. We selected 32 primary studies using predefined criteria and used a thematic analysis method for analyzing the extracted data.

**Results:**

Of the 1867 articles obtained, 32 were included in this review based on the predefined criteria. We identified 9 challenges that can affect the development of secure mHealth apps. These challenges include lack of security guidelines and regulations for developing secure mHealth apps (20/32, 63%), developers’ lack of knowledge and expertise for secure mHealth app development (18/32, 56%), lack of stakeholders’ involvement during mHealth app development (6/32, 19%), no/little developer attention towards the security of mHealth apps (5/32, 16%), lack of resources for developing a secure mHealth app (4/32, 13%), project constraints during the mHealth app development process (4/32, 13%), lack of security testing during mHealth app development (4/32, 13%), developers’ lack of motivation and ethical considerations (3/32, 9%), and lack of security experts’ engagement during mHealth app development (2/32, 6%). Based on our analysis, we have presented a conceptual framework that highlights the correlation between the identified challenges.

**Conclusions:**

While mHealth app development organizations might overlook security, we conclude that our findings can help them to identify the weaknesses and improve their security practices. Similarly, mHealth app developers can identify the challenges they face to develop mHealth apps that do not pose security risks for users. Our review is a step towards providing insights into the development of secure mHealth apps. Our proposed conceptual framework can act as a practice guideline for practitioners to enhance secure mHealth app development.

## Introduction

### Background

The use of mobile apps in health care has gained widespread adoption [1,2]. Lack of health professionals, especially in rural areas, is an excellent motivator for mobile health (mHealth) app adoption [3]. mHealth apps rely on the portability and context-sensitivity of mobile computing to improve access to health care services that are cost-effective, scalable, and pervasive [4]. Leveraging mHealth apps would improve access to health care services, lower the cost, and increase patients’ health awareness [5]. According to the World Health Organization, mHealth is defined as “medical and public health practice supported by mobile devices, such as mobile phones, patient monitoring devices, personal digital assistants (PDAs), and other wireless devices” [6]. There are several types of mHealth apps developed for health purposes ranging from general health apps such as decision, support, vitals, and reproductive health apps through fitness apps providing an activity tracker, nutrition tracker, and mindfulness [5]. The number of mHealth apps has grown massively following the launch of centralized mobile app repositories (ie, Google Play and Apple Store) in 2008. It has become easier for mobile developers to distribute their apps to a wide range of users [7]. Research2guidance, an organization for providing research and consultancy for digital health, reports that 78,000 new mHealth apps were added to apps stores in 2017. The report also showed that mHealth app downloads reached 3.7 billion, and the market revenue for digital health reached US $5.4 billion in 2017 [8].

The security of mobile apps in general and mHealth apps in particular has become one of the primary concerns since mobile apps are more vulnerable to attacks [9]. Most mobile apps collect, process, store, and transmit user and device data in and out of a device over various networks [5]. Compromising the confidentiality, integrity, and availability of such data would lead to severe consequences, including but not limited to compromised device data and leading to financial loss [10]. In mHealth apps, security becomes a significant concern due to health-critical data privacy and integrity [5,11]. An attack to falsify clinical measurements can lead to unnecessary care for patients as they think they are sicker than they actually are and can cause medical, legal, and social concerns [12].

Health professionals are increasingly relying upon health data collected via mHealth apps to make their decisions, such as dermatologic care [13], chronic illnesses management [14,15], and clinical practice [16]. Data manipulation can significantly impact treatment, causing serious results (eg, worsened morbidity or death) [17,18]. While health regulations and laws (ie, The US Health Insurance Portability and Accountability Act [HIPAA], European General Data Protection Regulation [GDPR]) strive to protect medical integrity and patients’ privacy by focusing on hospitals, doctors, and insurance firms, little attention has been paid to support mHealth app developers by providing them with suitable guidelines for developing secure apps [5,19].

A large part of mHealth app security relies on developers’ experience with designing and developing secure apps. We use the term developer in our research to refer to professionals who are engaged in engineering and development of mHealth apps. According to previous studies [1,12,15,20,21], most mHealth apps have not fully implemented mechanisms to protect health data. Studies have also claimed that mHealth developers may fail to appropriately implement basic security solutions such as authentication, encryption for data at rest, and encryption for data in transit. It is being recognized that it is critically important to thoroughly train mHealth app developers in implementing suitable security mechanisms to protect patients’ data from being stolen or compromised [12,20]. Hence, it is crucial to identify and synthesize the reported challenges of developing secure mHealth apps as a body of knowledge for research and practice. We have reviewed the relevant literature to determine the security challenges by focusing on developers rather than the solutions. Our research question for this literature review is: What are the challenges that developers of mHealth apps face with respect to implementing security?

This review’s primary contribution is identifying the challenges that hinder the development of secure mHealth apps, such as the lack of security guidelines and regulations for developing secure mHealth apps and developers’ lack of knowledge of and expertise with secure mHealth app development. This review’s results can be beneficial to researchers and practitioners (eg, mHealth app developers, managers, research engineers) for supporting research and development of emerging and next-generation, secure mHealth apps.

### Previous Work

The challenges for developing secure software have been receiving increasing attention in recent years. A review by Kanniah and Mahrin [22], which included 44 studies, identified the factors that influence secure software development practices. The study found that security skills, expertise, tools, and development time are among the factors that impact secure software development. The identified factors were classified into institutional context, people and action, project content, and software development process factors. Thomas et al [23] addressed the issues that security auditors face during application review for security bugs. The study recommend further support for the development process by providing security-related tools and effective communication tools for developer interactions. Further support for software developers has also been recommended by providing motivation (eg, reward or recognition) and providing solutions for technical challenges such as using third-party library issues. The authors recommended recruiting security experts within teams and make them available for answering questions. Raghavan et al [24] presented a model for achieving security during the software development lifecycle (SDLC). Their model suggests the following factors: security policy, management support, security-related training for developers, and development process control. Weir et al [25] studied the positive factors that enhance the development of secure software. The work identified the interventions that lead to achieving security by performing a threat model, organizing motivational workshops to engage team members, and continuous reminders for developers. The study also highlighted other interventions that need to be considered, such as component choice for security tools, performing static analysis, developer training, and performing penetration testing and code review.

Some studies also aimed to help mobile app developers develop secure apps by providing guidelines for the development process [6,26,27]. Given the increasing realization of the need to provide developers of mHealth apps with appropriate knowledge, training, and support for developing secure apps, there is a critical need to identify and analyze the challenges that prevent them from developing secure apps. Our findings would contribute to a body of knowledge about the challenges that mHealth app developers face with respect to security.

### Comparison With Prior Studies

Prior reviews [28,29] have focused more on investigating the security measures and technical solutions employed by developers. However, a few challenges were raised in [28,29]. Katusiime and Pinkwart [28] systematically reviewed privacy and usability issues and solutions in mHealth systems. The study considered developers’ lack of security knowledge and lack of a security framework as external factors that need to be considered. Another review by Marquez et al [29] was more on the security issues of telehealth systems. The study focused on classifying security (ie, attacks, vulnerabilities, weaknesses, and threats) and presenting security strategies (ie, detect attacks, stop or mitigate attacks, and react to attacks) of telehealth systems. Also, the study reported some security practices that need to be ensured, such as having a discussion about architectural styles (eg, security patterns) and engaging stakeholders during the development of an app. To the best of our knowledge, there is no systematic literature review (SLR) that explicitly investigates the challenges faced by mHealth app developers when implementing security for mHealth apps. Thus, we aimed to fill the gap and provide insights into the development of secure mHealth apps.

## Methods

**Figure 1:**
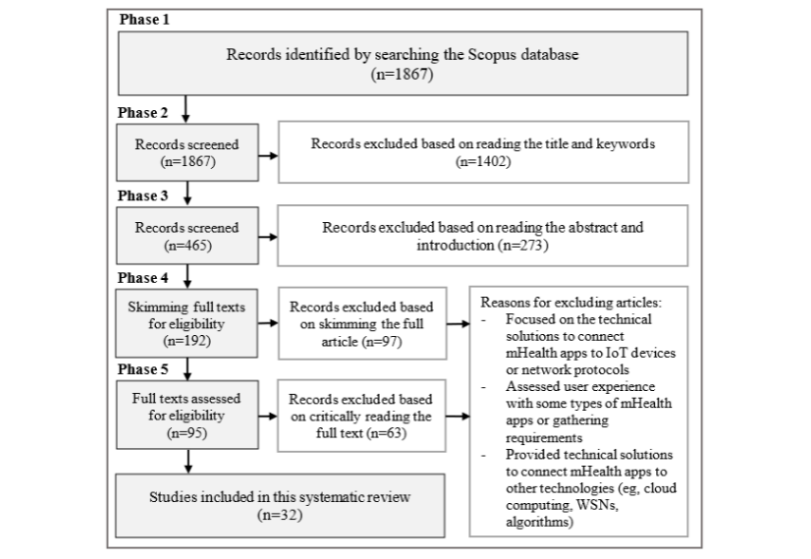
Flow diagram for the selection of articles. IoT: Internet of Things; mHealth: mobile health; WSN: wireless sensor network.

This research has been undertaken as an SLR. It is one of the most widely used research methods of evidence-based software engineering. An SLR provides a well-defined process for identifying, evaluating, and interpreting all available evidence relevant to particular research. We followed the guidelines of Kitchenham et al [30] to perform an SLR that involves 3 main phases: defining a review protocol, conducting the review, and reporting the review. In this section, we briefly describe the main components of the review protocol and its implementation. Our review protocol has 6 components, including research question, search strategy, data source, study selection process, inclusion and exclusion criteria, and data extraction and data synthesis. Figure 1 presents a flow diagram of the literature search and article selection results.

### Research Question

Our review’s objective was to identify and codify the challenges that hinder mHealth app developers from developing secure apps. This review’s findings would enable us to identify the potential gaps that need to be further investigated based on the developers’ perspectives.

### Search Strategy

We used the following strategies to form our search string: (1) identifying the major terms based on the study focus and the research question, (2) identifying all the possible keywords and related synonyms based on our experience and previous work, (3) using the Boolean “AND” to join major terms and the Boolean “OR” to join alternative terms and synonyms. Hence, our search string for this review was as follows: (“security” OR “insecure” OR “secure”) AND (“mobile health” OR “mobile healthcare” OR “mobile health-care” OR “mobile health care” OR “telehealth” OR “mhealth”).

### Data Source

We used the Scopus digital library as our primary search library as there are many successful examples of other researchers (eg, [30]) limiting their search to Scopus. The Scopus indexing system has the advantages of facilitating the formulated complex search string, being frequently updated, and keeping track of a large number of journals and conferences in software engineering studies. Furthermore, Scopus is an indexing database that provides name, keywords, and abstract for all published articles. Any pointed articles can be further searched and downloaded to review the whole article regardless of which database in which it actually exists.

### Study Selection Process

As illustrated in Figure 1, we followed several criteria to exclude studies in our SLR as detailed in the following sections.

#### Phase 1: Automatic Search

We ran our search string in the Scopus digital library. Thus, we retrieved a total of 1867 potential articles.

#### Phase 2: Title and Keyword-Based Selection

We carefully reviewed the title and keywords to decide whether each of the retrieved articles was relevant to our SLR. We retained the papers for the next inspection when we could not decide by reading the titles and keywords. Thus, we excluded 1402 articles and included 465 articles for the next phase.

#### Phase 3: Abstract and Introduction-Based Selection

We read the abstract and introduction for each article. This phase enabled us to include 192 articles and discard 273 articles.

#### Phase 4: Full Paper Scanning–Based Selection

We scanned the entire article to ensure that it was relevant to our SLR objective. Thus, we included 95 articles and excluded 97 articles.

#### Phase 5: Critical Review–Based Selection

We critically reviewed the included papers and excluded duplicates (eg, extended versions of the studies were included, and shorter versions were excluded). Thus, we excluded 63 articles and included 32 studies, referred to as S1 to S32. A list of the included papers is presented in Multimedia Appendix 1.

### Inclusion and Exclusion Criteria

For the purpose of this review, we applied predefined inclusion and exclusion criteria for paper selection. We included primary studies that focused on the development process of secure mHealth apps, studies written in English published from January 2008 to October 2020 since major app stores (Google Play and Apple Store) were launched in 2008, and peer-reviewed publications (ie, journals, conferences, workshops, and book chapters).

Besides excluding non-peer-reviewed studies (ie, lecture notes, summaries, panels, and posters) and studies that were not written in English, we excluded studies that contained irrelevant content for our review such as studies that focused on investigating technical solutions (eg, encryption methods, authentication mechanisms, access control) for mHealth apps; studies providing technical solutions to connect mHealth apps to Internet of Things (IoT) devices or cloud computing technology; studies that focused on sensor layers (eg, wireless sensor networks), developing algorithms, or network protocols for mHealth apps; studies that focused on mHealth app quality or gathering functional requirements; and studies that examined user experiences with some mHealth apps (eg, patient management apps).

### Data Extraction and Synthesis

**Figure 2:**
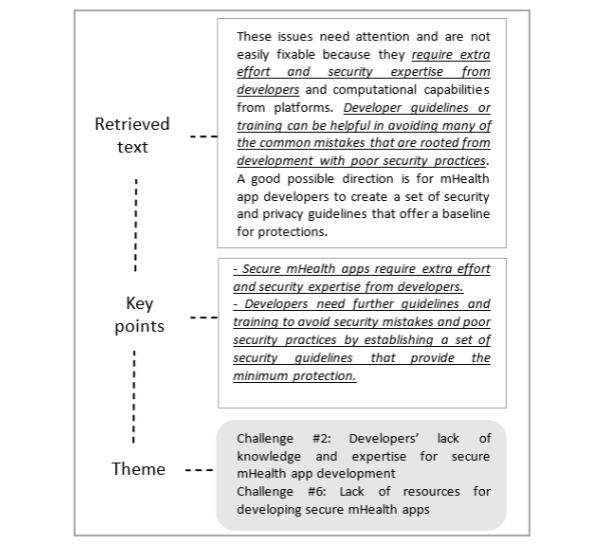
Example of the steps of applying the thematic analysis to the qualitative data. mHealth: mobile health.

We divided the extracted data into 2 categories: study characteristics and the challenges for developing secure mHealth apps. Our data extraction form is shown in Multimedia Appendix 2. We performed descriptive statistics to analyze the demographic data. To answer our research question, we used the Endnote tool to manage the bibliography and utilized Excel spreadsheets to extract and synthesize the data. We used thematic analysis, a qualitative analysis technique, to analyze and synthesize the extracted data to derive the results for this review [31]. We mainly followed the thematic analysis method’s 5 steps: (1) familiarizing oneself with the data, which involved trying to read and examine the extracted data items; (2) generating initial codes, which involved extracting the initial lists of challenges; (3) searching for themes, which involved trying to combine different initial codes generated from the second step into potential themes; (4) reviewing and refining themes, which involved checking the identified challenges from step 3 against each other to understand what themes had to be merged with others or dropped; and (5) defining and naming themes, which involved defining a name for each challenge. Figure 2 demonstrates an example that was taken from S4 [32] of how our final list of challenges was identified.

To further enhance our analysis, we developed a conceptual framework to present the correlation among the identified challenges. We followed the steps of Regoniel [33] to develop a conceptual framework that involves 4 steps: choose the topic, do a literature review, isolate the important variables, and generate the conceptual framework. It should be noted that the initial coding was done by the first author and was reviewed and revised (followed by a discussion wherever required) with 2 independent researchers, Dr Leonardo Iwaya and Dr Faheem Ullah, who are experts in the field of mHealth apps and doing SLR studies to avoid potential bias.

## Results

We now present the findings of our SLR. We classified the findings into demographic information, challenges for developing secure mHealth apps, and the conceptual framework for the identified challenges.

### Study Characteristics

In this subsection, we present the study characteristics based on the outlet (ie, journal, conference, or workshop) of the selected papers, as shown in Table 1.

Providing such information would be helpful for new researchers interested in conducting research in this particular area. We selected 32 primary studies for this review. The complete list of the reviewed articles is available in Multimedia Appendix 1. All selected studies mainly discussed the security aspects of mHealth apps. Table 1 shows the distribution, year of publication, and different outlets. It should be noted that our reviewed studies were published from 2012 to 2020. Of 32 studies, we noticed that 23 studies (72%) were published as journal papers; 7 studies (22%) were published in conferences, while 2 studies (6%) were published as workshop papers. Furthermore, we noticed that 11 studies (34%) were published in JMIR and JMIR mHealth and uHealth, and 2 studies were published at International Conference on Future Internet of Things and Cloud Workshops (2017, 2019).

**Table 1:** The number of selected studies published per year and their distribution by outlet.

Year	Journals, n	Conferences, n	Workshops, n
2012	1	1	0
2013	0	0	0
2014	5	1	0
2015	4	2	0
2016	2	0	0
2017	3	0	1
2018	4	0	0
2019	4	1	1
2020	0	2	0

### Challenges With Developing Secure mHealth Apps

This subsection reports the results based on our analysis to answer the study research question: “What are the challenges that developers of mHealth apps face with respect to implementing security?” Our analysis identified 9 challenges (referred to as C1 to C9) that hinder app developers from developing secure mHealth apps. The identified challenges were ordered based on their frequency within the reviewed studies. Table 2 illustrates the identified challenges, the key points that led us to consider them from the reviewed studies, and the frequency of each challenge.

**Table 2:** Challenges with developing secure mobile health (mHealth) apps (identified from 32 studies).

Challenge number and description	Key points from reviewed studies	Frequency, n (%)
C1. Lack of security guidelines and regulations for developing secure mHealth apps	Lack of security guidelines, regulations, direct laws about the security requirements, secure designing, security testing, security features that need to be employed in mHealth apps (S4 [32], S5 [17], S6 [34], S7 [35], S10 [36], S12 [9], S13 [37], S15 [38], S16 [19], S20 [39], S22 [20], S23 [1], S26 [40], S29 [41], S31 [42]); lack of framework or standards (eg, standardized policies and methodologies to ensure the security standards are met) for developing secure mHealth apps (S2 [43], S3 [12], S29 [41], S31 [42]); lack of compliance with the available guidance and/or standard (S25 [44], S29 [41]); challenges for the developers to deal with legal obligations, policies, and procedures (S32 [4])	20 (63)
C2. Developers’ lack of knowledge of and expertise with secure mHealth app development	Insufficient knowledge of software developers about the security risks of mHealth apps (S12 [9], S17 [45], S18 [46], S27 [47]); lack of developers’ security awareness (eg, towards the potential threats of mHealth apps; S3 [12], S9 [11], S14 [18], S21 [15], S28 [48], S32 [4]); developers’ lack of knowledge towards secure coding practices, using secure APIs^a^, and utilizing up-to-date libraries (S18 [46]) or secure third-party services by mHealth app developers that could misuse users' health data (S1 [21], S11 [5], S19 [2], S24 [49]); developers’ lack of knowledge towards utilizing security measures (eg, TLS^b^ security for servers, proper protection for user passwords) of mobile devices (S3 [12], S8 [50], S22 [20], S25 [44]); lack of experience in secure software development for developers (S4 [32]); lack of auditing security knowledge and review what knowledge they have (S25 [44])	18 (56)
C3. Lack of stakeholders’ involvement during mHealth app development	Lack of stakeholders’ participation during the development lifecycle of mHealth apps (S5 [17], S10 [36], S20 [39], S29 [41], S30 [51]); lack of security understanding by health professionals when they engage in the development process causing poor elicitation of security requirements (S5 [17])	6 (19)
C4. No or little attention by developers towards the security of mHealth apps	Developer' assumption that users are not concerned about security (S32 [4]); security is not developers’ concern (S11 [5], S21 [15]); security issues should be resolved by the testers (S32 [4]); developers with no security focus skip all security measures (S18 [46]); developers are not considering secure design principles and privacy guidelines (S31 [42])	5 (16)
C5. Lack of financial resources for developing secure mHealth apps	No/low budget assigned for employing security measures (S32 [4]); unavailability of security tools (S32 [4]); developers lack training about developing secure mHealth apps (S4 [32], S5 [17]); lack of research and development efforts to facilitate developing secure mHealth apps (S14 [18])	4 (13)
C6. Time constraints during mHealth app development process	Rushing to market, which leaves vulnerabilities in mHealth apps (S18 [46], S26 [40], S32 [4]); the long process of gaining consent or approving the development choices of the developers (S7 [35])	4 (13%)
C7. Lack of security testing during mHealth app development	Lack of security testing (S32 [4]); lack of proper security testing (eg, vulnerability scan) for mHealth apps (S6 [34], S18 [46], S23 [1])	4 (13)
C8. Developers lack motivation and ethical considerations	Lack of motivations for developers during the development process of mHealth apps (S27 [47]); developers lack ethics during the development process of mHealth apps (S10 [36], S30 [51])	3 (9)
C9. Lack of security experts’ engagement during mHealth app development	Lack of collaboration and discussion with security experts from the beginning of the development lifecycle of mHealth apps (S18 [46], S32 [4])	2 (6)

^a^APIs: application programming interfaces.

^b^TLS: transport security layer.

#### C1: Lack of Security Guidelines and Regulations for Developing Secure mHealth Apps

Security guidelines refer to a set of suggested actions or recommendations for things to do or avoid during software development [52]. Security guidelines help app developers, mostly inexperienced, adopt effective security practices and write secure codes. They contain accessible information, properly layered and searchable, with good coverage of all security aspects (eg, cryptography, handling user input and privileges [26]). It would be ideal to clarify that there are numerous security guidelines for ensuring mobile app security (eg, Open Web Application Security Project [OWASP]). According to Nurgalieva et al [53], the available security guidance for developing secure mHealth apps can be categorized into (1) guidelines, recommendations, or principles; (2) app development practices (ie, applied security mechanisms) to ensure mHealth security; and (3) models of user behavior and preferences related to security or privacy. Such guidelines (eg, GDPR) have had the effect of raising awareness and establishing a minimal set of expectations. However, they do not address the issue of the development of systems that meet privacy and security requirements [53]. Additionally, Assal and Chiasson [54] indicated that security guidelines do not exist or are not mandated by the companies or that developers might lack the ability or proper expertise to identify vulnerabilities despite having general security knowledge. Our reviewed studies, including S3 [12], S4 [32], S12 [9], and S20 [39], have pointed out a general lack of security guidelines for developing secure mHealth apps. Zubaydi et al [9] called for effective guidelines that can help developers build secure mHealth apps (S12 [9]). Even though there are guidelines to protect health data (ie, HIPAA), they do not provide specific instructions for developing secure mHealth apps. Furthermore, it has also been claimed that there is a lack of security frameworks, standards, compliance checklists, and regulations (S22 [20], S18 [46], S13 [37], S20 [39], S2 [43], S9 [11]). Legal restrictions (ie, obtaining security certification) ensure that mHealth app development organizations are not developing vulnerable mHealth apps (S11 [5], S12 [9]).

#### C2: Developers’ Lack of Knowledge of and Expertise With Secure mHealth App Development

The security knowledge of mobile app developers plays a significant part in developing secure mHealth apps. Lack of security knowledge would result in creating an insecure app that may leak health-critical data to attackers. The reviewed studies indicated that mHealth app developers do not have enough security education covering important security aspects. Consequently, developers follow insecure programming practices (eg, employing improper security solutions; S22 [20], S19 [2], S25 [44]) or improper handling for mHealth app permissions (S23 [1]). Furthermore, developers’ lack of security knowledge may lead to incorrect security choices when attaching a particular device with mHealth apps (eg, tracking device that helps monitor user behavior; S11 [5], S12 [9], S18 [46]) or integrating an app with other systems (S13 [37]). Making an incorrect security decision may allow health apps to share health-critical data with other mobile apps, untrusted apps, or external hosts (S12 [9]). mHealth app developers may make security-centric decisions based on their best assumption or strategies (S24 [49]). Thamilarasu and Lakin (S18 [46]) conducted a vulnerability scan that revealed 248 vulnerabilities in the top 15 Android-based mHealth apps. The study revealed that the 3 most common vulnerabilities were not errors in systems, but instead, errors in developers' choices (ie, selecting a suitable cipher, choice of permissions to request on a mobile device). The study concluded that most vulnerabilities could have been prevented through proper coding and secure engineering practices.

Keeping in mind that the threat landscape is changing rapidly, dealing with the volatile environment requires developers to keep their security knowledge sharp. Even security experts need to update their knowledge [55]. Despite the fact that mHealth app vulnerabilities are frequently announced in security-relevant knowledge banks (eg, National Vulnerabilities Database, data breach reports) to advise developers, for some reason (ie, difficult to use), these security alerts are not followed or ignored. As a result, unfixed bugs might allow attackers to perform malicious activities (eg, illegally access health-critical data by exploiting sensor permissions, enabling them to extract data or transfer malware to an app [56]). Announcements of identified security bugs are one way of encouraging mHealth developers to keep up to date with the threat landscape. Müthing et al [2] and Dehling et al [18] indicated that mHealth app developers use out-of-date security measures (S14 [18], S19 [2], S23 [1]). As a result, some mHealth apps even have previously exposed security errors (S23 [1]). Despite the realization of the importance of keeping mHealth developers aware of the latest security issues, there is a little evidence that developers get regular formal security training to maintain their security knowledge (S24 [49]). Lack of auditing among developers to maintain and review their security knowledge can create a knowledge gap and lead to out-of-date security knowledge (S25 [44]).

#### C3: Lack of Stakeholders’ Involvement During mHealth App Development

Involving stakeholders in security requirement engineering is being recognized as key to software success and getting effective and impactful outcomes [22]. Indeed, stakeholders’ involvement contributes to the elicitation and specification of security requirements of the developed software. Yet, it is difficult, as developers would first exert significant effort to understand the complexity of a problem domain [57]. Also, more time and resources would be required. For mHealth apps, developers should refer to stakeholders (eg, medics, patients) throughout the development process to ensure that the technology meets their needs (S10 [36], S30 [51]). Further, stakeholders need to be involved earlier in the development process of mHealth apps. However, development practices often include clinicians and experts but more rarely involve the target audience until evaluation (S29 [41]). At the same time, it would be challenging for some stakeholders to have an understanding of security due to their capabilities when engaging them in the development process. As a result, this causes poor elicitation of security requirements (S5 [17], S20 [39]).

#### C4: Lack of Financial Resources for Developing Secure mHealth Apps

The development process of mHealth apps can be supported by using security resources to enhance secure mHealth app development. Lack of necessary resources, such as technology, is a challenge that can directly impact developing secure mHealth apps. For example, security tools (eg, Zed Attack Proxy, Android Debug Bridge, Codified Security, White Hat Security, and Quick Android Review Kit) are resources to facilitate writing secure code and testing apps during the development process. They help developers catch errors that they might be unaware of and adjust their code accordingly before releasing an app. Wurster and van Oorschot [58] argued that not all software developers are security experts, and there is a need to use suitable security tools during a development project. Security tools for mobile apps have received a lot of attention from researchers. A security tool called FixDroid [59] can show warning messages with recommendations to fix errors during the coding phase. It has proven to be effective in improving the security of the written code, is limited to Android app developers, and is not widely known.

Similarly, software libraries can be used as supportive resources to facilitate the software development process. Such libraries help developers reuse specific code for certain goals and support access to hardware and software that might be needed. Yet, it can be challenging for developers to know which library to trust while developing mHealth apps. There can be a risk of data leakage by using untrusted libraries (S16 [19], S13 [37]). Some libraries, especially the open-source libraries, may collect data about users without developers being aware of it, leading to data privacy breaches [60]. Furthermore, using untrusted third-party libraries to integrate mHealth apps with electronic health records can result in attackers gaining unauthorized access to patients’ data (S13 [37]).

Older versions of security resources (ie, tools and libraries) also contain known vulnerabilities (S18 [46]). Most of the security resources are often updated to address security-related issues and introduce new functions; hence, it is important to be aware of and use the latest security tools and libraries. Therefore, developers’ security knowledge of the adopted security resources can significantly impact the developed app’s security. Besides being aware of the relevant security resources, it can be difficult for developers to learn to use them within the time and resources available for a project (S25 [44], S17 [45]).

#### C5: No or Little Attention by Developers Towards the Security of mHealth Apps

Incorporating security should ideally be considered throughout SDLC from requirement analysis to the deployment phase [61]. In fact, addressing security at later stages of app development or after app release in the form of security patches can be a costly exercise and can introduce new vulnerabilities [62]. Studies, such as S11 [5], S21 [15], and S31 [42], found that mHealth app developers pay little or no attention to the security of mHealth apps. This issue can be seen for a few reasons, including (1) developer' assumption that users are not concerned about security, (2) developers’ assumption that security should be handled by app testers, and (3) developers with no security focus would even skip all security measures to resolve other quality attributes including usability and performance (S18 [46], S32 [4]). Therefore, it is important to come up with effective mechanisms for overcoming developers’ lack of attention towards security.

#### C6: Time Constraints During mHealth App Development Process

Due to business pressures (eg, rushing to market), delivering an app on time tends to be the main aim mHealth apps developers try to satisfy for customers and avoid extra costs. High workload and tight timeframes require mHealth app developers to put more effort in meeting functional requirements as a primary task (S18 [46], S26 [40], S32 [4]). It also affects their attitude and behavior towards addressing security (eg, underestimating risks, assuming attackers will not realize the weaknesses) and dealing with security after releasing an app [61]. This approach leads to insecure mHealth apps, increases the cost, and introduces new vulnerabilities after fixing the existing vulnerabilities [63]. It is estimated that the cost can be 30 to 100 times more expensive to retrofit security compared with incorporating security from the beginning [64]. Besides, the speed of delivering apps will not allow team members to share and convey security knowledge among mHealth app developers [65]. Furthermore, the long process of gaining consent or manager approval of the developers’ choices can be an issue (S7 [35]). As a result, this lengthens the process of getting their opinion on a specific task. Hence, this leads to skipping security issues that need to be fixed.

#### C7: Lack of Security Testing During mHealth App Development

Security testing is one of the essential phases of the mHealth app development lifecycle. Security testing helps determine the quality of apps by ensuring all the security requirements are met. Security testing for mHealth apps, in particular, will help figure out how an app will react against different attacks (eg, unauthorized access to health data, tampering with health data, or reporting invalid health data to health professionals; S11 [5]). Security testing of mHealth apps can be overlooked since it can be a challenging task for developers. Several factors can affect performing security testing, including the absence of security testing tools, lack of effective and well-known testing guidelines, cost of performing app testing by a third-party organization, or lack of a security expert within a software development organization (S23 [1], S18 [46], S6 [34]). Consequently, this would release mHealth apps without conducting security testing, leaving an app at high risk [66]. Wurster and van Oorschot [58] indicated that security testing is not a first-choice task for developers, and their main job is completing the required features.

#### C8: Lack of Security Experts’ Engagement During mHealth App Development

A security expert, security leader, or security champion within an organization plays a vital role during the mHealth app development process (S7 [35]). Besides the development activities, they direct mHealth app developers on secure development practices and perform a security review to ensure their code does not have security defects. A security expert can encourage developers to achieve security goals and educate other developers about the potential threats and solutions (S14 [18]). Lack of security experts within a software development team can lead to failures in applying proper security controls by mHealth app developers. Besides, the lack of availability of security experts would be a challenge for developers (S7 [35]). As a result, there is a lack of constructive feedback that prevents developers from (1) acquiring security knowledge, (2) gaining hands-on experience, and (3) developing apps that are secure by design.

#### C9: Developers’ Lack of Motivation and Ethical Considerations

Motivation refers to the driving force behind all the actions of developers during development. It has been recognized as a critical success factor for software projects. Motivation can be seen differently based on developers and an organization’s size [67]. The research on security practices indicates that many security incidents are mainly caused by human rather than technical failure [68]. Developers with low motivation were found to be one of the most frequently cited causes of software development project failures [69]. Xie et al [70] presented the reasons that make software developers make security errors. The study concluded that most software developers have a “not my problem” attitude, which indicates that software developers are the source of security errors due to their attitudes and behaviors. In particular, in mHealth app development, studies such as S10 [36], S27 [47], and S30 [51] reported that developers’ lack of motivation and ethical considerations is a challenge that hinders developing secure mHealth apps.

### Conceptual Framework

**Figure 3:**
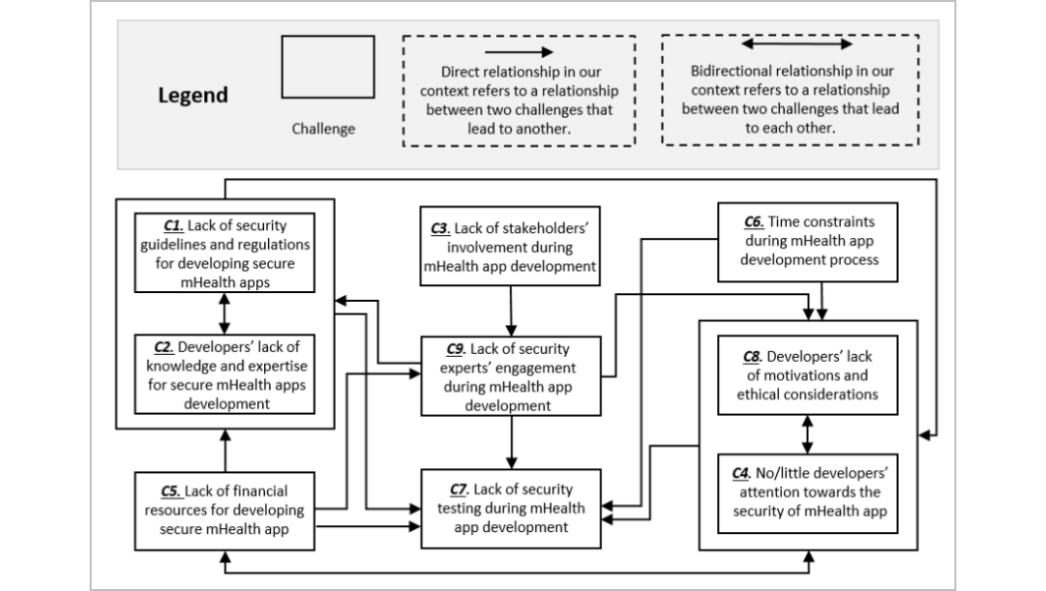
A conceptual framework for correlating the challenges in developing secure mHealth apps.

Based on our analysis of the extracted data, we propose a conceptual framework, as in Figure 3, that represents the challenges for developing secure mHealth apps. Jabareen [71] defined a conceptual framework as “a network, or a plane of interlinked concepts that together provide a comprehensive understanding of a phenomenon or phenomena.” Figure 3 presents a conceptual framework for correlating the identified challenges.

Based on the results of Table 2, we identified the most critical challenges for developing secure mHealth apps. Critical challenges can be determined if a specific challenge has a frequency of ≥50% of the selected studies. This criterion has been used by other researchers in different domains [72]. As in Table 2, the frequencies are shown for each challenge in the reviewed studies. By using this criterion, we concluded that there are 2 main critical challenges: lack of security guidelines for developing secure mHealth apps (20/32 studies, 63%) and developers’ lack of security knowledge and expertise for secure mHealth app development (18/32 studies, 56%).

Despite the fact that other challenges were given less attention by the reviewed studies (ie, 19% for C3, 16% for C4, 13% for C5-C7, 9% for C8, and 6% for C9), some challenges have a direct relationship with other challenges as we indicated earlier (eg, poor security decisions during mHealth app development are related to insufficient security knowledge by developers). Consequently, there will be an impact on the development process of mHealth apps. Therefore, we believe identifying these challenges would help mHealth app development organizations evaluate their security practices and readiness in implementing security in mHealth app projects.

## Discussion

### Principal Findings

While mHealth apps enable health care services, the security of end users’ health data remains a challenge. This review aimed to identify the challenges that prevent development of secure mHealth apps based on the existing literature. We identified 9 challenges based on the analysis of the data extracted from 32 articles. The identified challenges include (1) lack of security guidelines and regulations for developing secure mHealth apps, (2) developers’ lack of knowledge of and expertise with secure mHealth app development, (3) lack of stakeholders’ involvement during mHealth app development, (4) no or little attention by developers towards the security of mHealth apps, (5) lack of resources for developing secure mHealth apps, (6) project constraints during the mHealth app development process, (7) lack of security testing during mHealth app development, (8) developers’ lack of motivation and ethical considerations, and (9) lack of security experts’ engagement during mHealth app development. We noticed from the literature that there is an emphasis on presenting the security issues of mHealth apps and how they can be resolved (eg, presenting security framework, providing secure mHealth app development recommendations, evaluating the security for existing mHealth apps). However, little attention has been given to the human factor during the development process of mHealth apps (ie, nontechnical solutions). Hence, it would be critical to recognize the security challenges that mHealth app developers face during the development process.

Sufficient security knowledge for mHealth app developers is one of the key factors that would help develop secure apps. Security knowledge can be discussed as the type of required security knowledge and the sources of acquiring that knowledge. According to Barnum and McGraw [52], there are 7 security knowledge categories for developing secure software, including knowledge of principles, guidelines, rules, attack pattern, vulnerability, exploit, and historical risk. While the presented set of security knowledge provides a strong foundation for enhancing security, it would be a bit challenging for developers since security knowledge is scattered all around. By considering security knowledge of vulnerabilities as an example, attackers can find a single vulnerability to exploit an app (ie, launching an attack). In contrast, developers should be aware of all security vulnerabilities and apply proper security measures and patches, which can be a daunting task. mHealth apps, more specifically, are connected to IoT devices, which makes securing the apps a challenge. Sikder et al [56] indicated that attackers could illegally access health data by exploiting sensors’ permissions, which could enable them to extract data and transfer malware to an app. Therefore, further support for mHealth app developers' security knowledge is needed to cope with the rapid changes in security knowledge.

Likewise, using trusted sources (ie, tools and libraries) would be challenging for developers to be aware of their secure usage. So, we suggest further required improvement to facilitate mHealth app developers’ jobs by exploring the list of trusted sources. Identifying trusted sources with their policies, terms, and conditions of usage and the proper ways of receiving updates would help mHealth app developers to develop secure apps. At the same time, this approach would help disseminate and provide security knowledge for mHealth app developers through trusted sources.

### The Role of Security Experts Within mHealth App Development

“A critical challenge facing software security today is the dearth of experienced practitioners” [52]. A report by Ponemon Institute showed there is a dearth of security experts in mobile app development. Only 41% of the participants indicated that their organizations had sufficient security expertise [64]. Hence, having a security expert can be a strategic advantage for an organization. The role of security experts is quite crucial in developing secure mHealth apps. We conclude from the conceptual framework (ie, Figure 3) that a lack of security experts is already linked to most challenges. Without security experts on a team, the required security knowledge will be missing (ie, what security guidelines need to be followed, what security tools are available to be utilized, and which libraries can be trusted). As a result, developers’ security knowledge would remain insufficient. Lack of security experts within mHealth app development organizations can lead to poor coding practices, rushing to deliver an app without even performing security testing. Furthermore, collaboration and social interactions with security experts and other team members would significantly impact security. As a result, removing the boundaries and stimulating common interests, in turn, support exchanging knowledge and ideas [67]. Also, it is good practice to exchange security knowledge, leverage that knowledge within the project, and acquire new knowledge.

### Importance of Security Knowledge and Expertise to Develop Secure mHealth Apps

Our analysis shows that developers’ lack of security knowledge and expertise for secure mHealth app development is correlated with most of the identified challenges. For instance, developing secure mHealth apps requires good knowledge about security guidelines, security tools, and the trusted libraries (ie, awareness of how, when, and why they should utilize them). It is worth mentioning that development of secure mHealth apps has become complex and challenging. mHealth apps require connection with external sensors or devices (eg, wearable devices, implantable devices) [56]. Nevertheless, providing the required learning resources can be underestimated by mHealth app development organizations [65]. Thus, organizations are required to provide security material to allow developers to learn to connect mHealth apps with emerging technologies (ie, IoT). Providing resources to support secure mHealth app development would contribute to filling the security knowledge gap and help open developers’ mindset to security errors that need to be avoided [55].

### Future Work

The results of our review enabled us to propose the following areas that warrant future research on the secure development of mHealth apps.

#### Challenges With and Practices of Developing Secure mHealth Apps With Real-World Practitioners

In this review, we identified the challenges that hinder developing secure mHealth apps based on SLR. We plan to conduct an empirical study to investigate the challenges with real-world practitioners to validate our results. The planned future research would enable us to compare the identified challenges identified from the literature with real-world practices for better understanding. Further, we aim to study the practices that real-world practitioners use to overcome the identified challenges. As a consequence, this would allow us to define which challenges are correlated with which practices. Hence, identifying the challenges and practices would help us to extend the current conceptual framework and provide a body of knowledge for secure mHealth app development.

#### Developers’ Motivations and Ethical Considerations for Developing Secure mHealth Apps

Since motivations and ethical considerations play an essential role in the secure mHealth app development process, we assert that there is a need to conduct an empirical study to understand developers’ motivational factors and what inspires them to ensure the security of mHealth apps (eg, security leaders, reward, recognition, career path, or promotion). Such a study can be further investigated by collecting quantitative data (eg, hypothesis testing) or qualitative data. This would create a better understanding and help mHealth app development organizations to realize and focus on the motivational factors.

### Limitations

One of the potential threats for our SLR can be missing or excluding relevant studies. To mitigate this threat, we used Scopus library as our data source. Scopus is considered the largest indexing system that provides the most comprehensive search engine, among other digital libraries [73]. Scopus enabled us to get a reasonable number of studies (1867 articles). Furthermore, we tested our search string based on the pilot search to improve it and reach the relevant studies for this review. We selected the studies based on predefined inclusion and exclusion criteria. However, including and excluding studies can be impacted by researchers’ subjective judgement. To mitigate this threat, the reasons for excluding the papers were recorded and reviewed by 2 independent researchers (who were previously mentioned).

Our research can be influenced by the researcher’s bias in extracting data from the reviewed studies, which may negatively affect the findings. To overcome this threat, we extracted data based on a predefined data extraction form (see Multimedia Appendix 2). To mitigate the researcher’s bias in data extraction and synthesis, the second author and the 2 independent researchers randomly verified the key points and themes derived by the first author through discussions.

### Conclusion

This review was motivated by the growing amount of attention paid to mobile apps, particularly mHealth apps. We aimed to analyze and synthesize the literature to identify the challenges that hinder mHealth app developers from developing secure apps. Our review followed an SLR approach and selected 32 studies that we believed were relevant to our study. We identified and discussed 9 challenges faced by mHealth app developers to develop secure apps. We also provided a conceptual framework for the identified challenges and presented several challenges linked to the body of knowledge found in this literature review. Our findings can be valuable for researchers and practitioners (eg, mHealth app developers, managers) to support research and development of secure mHealth apps. For researchers, this review can help formulate and test hypotheses. Furthermore, ideal and innovative solutions can be proposed to address these challenges. For practitioners, our review can help understand the existing challenges for developing secure mHealth apps from the literature. This would help resolve these challenges at the early stages of the mHealth app development process.

## Appendix

Multimedia Appendix 1 List of the reviewed studies.

Multimedia Appendix 2 Data extraction form.

## Abbreviations

GDPR: General Data Protection Regulation
HIPAA: Health Insurance Portability and Accountability Act
IoT: Internet of Things
mHealth: mobile health
OWASP: Open Web Application Security Project
SDLC: software development lifecycle
SLR: systematic literature review
