# Supplementary material for: Challenges With Developing Secure Mobile Health Applications: Systematic Review
Source: JMIR Mhealth Uhealth. 2021 Jun 21;9(6):e15654. doi: 10.2196/15654 (PMC8277314; doi:10.2196/15654)
Supplement: Multimedia Appendix 1 [file mhealth_v9i6e15654_app1.docx]

List of the reviewed studies

| ID | Author(s) | Title | Venue | Pub. Year |
| --- | --- | --- | --- | --- |
| S1 | S. Gejibo, F. Mancini, K. A. Mughal, R. A. B. Valvik, and J. Klungsøyr | Secure data storage for mobile data collection systems | International Conference on Management of Emergent Digital EcoSystems | 2012 |
| S2 | D. D. Luxton, R. A. Kayl, and M. C. Mishkind | MHealth data security: The need for HIPAA-compliant standardization | Telemedicine and e-Health | 2012 |
| S3 | R. Adhikari, D. Richards, and K. Scott, | Security and privacy issues related to the use of mobile health apps | 25th Australasian Conference on Information Systems | 2014 |
| S4 | D. He, M. Naveed, C. A. Gunter, and K. Nahrstedt | Security Concerns in Android mHealth Apps | Annual Symposium proceedings / AMIA Symposium. | 2014 |
| S5 | T. L. Lewis and J. C. Wyatt | mHealth and mobile medical apps: a framework to assess risk and promote safer use | Journal of medical Internet research | 2014 |
| S6 | S. Becker, T. Miron-Shatz, N. Schumacher, J. Krocza, C. Diamantidis, and U.-V. Albrecht | mHealth 2.0: experiences, possibilities, and perspectives | JMIR mHealth and uHealth | 2014 |
| S7 | I. Mergel | The Long Way From Government Open Data to Mobile Health Apps: Overcoming Institutional Barriers in the US Federal Government | JMIR mHealth and uHealth | 2014 |
| S8 | S. Arora, J. Yttri, and W. Nilsen | Privacy and security in mobile health (mHealth) research | PubMed, Alcohol Research | 2014 |
| S9 | Y. Cifuentes, L. Beltrán, and L. Ramírez | Analysis of Security Vulnerabilities for Mobile Health Applications | The Seventh International Conference on Mobile Computing and Networking | 2015 |
| S10 | A. Carter, J. Liddle, W. Hall, and H. Chenery | Mobile phones in research and treatment: ethical guidelines and future directions | JMIR mHealth and uHealth | 2015 |
| S11 | K. Knorr and D. Aspinall | Security testing for Android mHealth apps | IEEE 8th International Conference on Software Testing, Verification and Validation Workshops, | 2015 |
| S12 | F. Zubaydi, A. Saleh, F. Aloul, and A. Sagahyroon | Security of mobile health (mHealth) systems | IEEE 15th International Conference on Bioinformatics and Bioengineering | 2015 |
| S13 | A. Landman, S. Emani, N. Carlile, I. D. Rosenthal, S. Semakov, J. D. Pallin | A Mobile App for Securely Capturing and Transferring Clinical Images to the Electronic Health Record: Description and Preliminary Usability Study | JMIR mHealth and uHealth | 2015 |
| S14 | T. Dehling, F. Gao, S. Schneider, and A. Sunyaev | Exploring the Far Side of Mobile Health: Information Security and Privacy of Mobile Health Apps on iOS and Android | JMIR mHealth and uHealth | 2015 |
| S15 | J. Hsu, D. Liu, Y. M. Yu, H. T. Zhao, Z. R. Chen, J. Li | The top Chinese mobile health apps: A systematic investigation | Journal of Medical Internet Research | 2016 |
| S16 | D. Kotz, C. A. Gunter, S. Kumar, and J. P. Weiner | Privacy and Security in Mobile Health: A Research Agenda," | Computer, vol. 49, pp. 22-30 | 2016 |
| S17 | M. Zens, P. Woias, N. Südkamp, and P. Niemeyer | Back on track: cruciate ligament study via smartphone: Practical example of possibilities for the Apple ResearchKit | Arthroskopie, vol. 29 | 2016 |
| S18 | G. Thamilarasu and C. Lakin | A security framework for mobile health applications | 5th International Conference on Future Internet of Things and Cloud Workshops | 2017 |
| S19 | J. Müthing, T. Jäschke, and M. C. Friedrich | Client-Focused Security Assessment of mHealth Apps and Recommended Practices to Prevent or Mitigate Transport Security Issues | JMIR mHealth and uHealth | 2017 |
| S20 | M. Bradway, C. Carrion, B. Vallespin, O. Saadatfard, E. Puigdomènech, M. Espallargues | mHealth Assessment: Conceptualization of a Global Framework | JMIR mHealth and uHealth | 2017 |
| S21 | T. Mabo, B. Swar, and S. Aghili | A vulnerability study of Mhealth chronic disease management (CDM) applications (apps) | Advances in Intelligent Systems and Computing | 2018 |
| S22 | A. Papageorgiou, M. Strigkos, E. Politou, E. Alepis, A. Solanas, and C. Patsakis, | Security and Privacy Analysis of Mobile Health Applications: The Alarming State of Practice | IEEE Access | 2018 |
| S23 | M. Hussain, A. A. Zaidan, B. B. Zidan, S. Iqbal, M. M. Ahmed, O. S. Albahri, et al. | Conceptual framework for the security of mobile health applications on Android platform | Telematics and Informatics | 2018 |
| S24 | L. Hutton, B. A. Price, R. Kelly, C. McCormick, A. K. Bandara, T. Hatzakis, et al. | Assessing the privacy of mhealth apps for self-tracking: heuristic evaluation approach | JMIR mHealth and uHealth | 2018 |
| S25 | M. Aliasgari, M. Black, and N. Yadav | Security vulnerabilities in mobile health applications | IEEE Conference on Application, Information and Network Security | 2019 |
| S26 | Y. M. Al-Sharo | Networking issues for security and privacy in mobile health apps | International Journal of Advanced Computer Science and Applications | 2019 |
| S27 | L. Parker, V. Halter, T. Karliychuk, and Q. Grundy | How private is your mental health app data? An empirical study of mental health app privacy policies and practices | International Journal of Law and Psychiatry | 2019 |
| S28 | M. Srivastava and G. Thamilarasu | MSF: A comprehensive security framework for mhealth applications | International Conference on Future Internet of Things and Cloud Workshops | 2019 |
| S29 | T. Wykes and S. Schueller | Why reviewing apps is not enough: Transparency for trust (T4T) principles of responsible health app marketplaces | Journal of Medical Internet Research | 2019 |
| S30 | T. Wykes, J. Lipshitz, and S. M. Schueller | Towards the Design of Ethical Standards Related to Digital Mental Health and all Its Applications | Current Treatment Options in Psychiatry | 2019 |
| S31 | J. Muchagata, S. Teles, P. Vieira-Marques, D. Abrantes, and A. Ferreira | Dementia and mHealth: On the Way to GDPR Compliance | Communications in Computer and Information Science | 2020 |
| S32 | B. Aljedaani, A. Ahmad, M. Zahedi and M. Ali Babar | An Empirical Study on Developing Secure Mobile Health Apps: The Developers’ Perspective | Asia-Pacific Software Engineering Conference | 2020 |
