# Supplementary material for: Challenges With Developing Secure Mobile Health Applications: Systematic Review
Source: JMIR Mhealth Uhealth. 2021 Jun 21;9(6):e15654. doi: 10.2196/15654 (PMC8277314; doi:10.2196/15654)
Supplement: Multimedia Appendix 2 [file mhealth_v9i6e15654_app2.docx]

Data Extraction Form

| # | Data item | Description |
| --- | --- | --- |
| D1 | Author(s) |  |
| D2 | Year | Demographic data |
| D3 | The Name of Publication Venue | Demographic data |
| D4 | Title |  |
| D5 | Publication Type (i.e., journal, conference, workshop) | Demographic data |
| D6 | Challenges that hinder developing secure mobile health apps. | RQ |
